# Pigmentary abnormality without significant drusen as a risk factor for late age-related macular degeneration

**DOI:** 10.1038/s41598-022-04798-8

**Published:** 2022-01-14

**Authors:** Junwon Lee, Hyun Goo Kang, Hae Rang Kim, Christopher Seungkyu Lee, Min Kim, Sung Soo Kim, Suk Ho Byeon

**Affiliations:** 1 Department of Ophthalmology, Institute of Human Barrier Research, Gangnam Severance Hospital, Yonsei University College of Medicine, Seoul, South Korea; 2 Department of Ophthalmology, Eye Hospital, Severance Hospital, Institute of Vision Research, Yonsei University College of Medicine, Yonsei-ro 50-1, Seodaemun-Gu, Seoul, 03722 South Korea

**Keywords:** Eye diseases, Risk factors

## Abstract

We investigated the incidence and risk factors of late age-related macular degeneration (AMD) in the fellow eye (FE) without significant drusen of patients with unilateral exudative macular neovascularization (MNV). In this retrospective study, 241 eligible patients who were followed-up for more than 3 years were enrolled. We analyzed the incidence and hazard ratios (HRs) of late AMD in the FE according to demographic and ophthalmologic variables. Hypopigmentation on color fundus photography (CFP) corresponds to shallow irregular RPE elevation (SIRE), so-called “double-layer sign” and/or “attenuation or disruption of RPE and/or ellipsoid zone” on OCT. The 5-year incidence of FE exudative MNV conversion was 8.6%. The 5-year incidence of FE exudative MNV of large hypopigmentation (≥ 0.5 disc area; DA) and small hypopigmentation (< 0.5 DA) on CFP, and SIRE (≥ 1000 µm) and small RPE elevation (< 1000 µm) on OCT were 36.2%, 14.2%, 55.0%, and 15.6%, respectively. The multivariate Cox proportional hazard model revealed that large hypopigmentation, small hypopigmentation, SIRE, and small RPE elevation showed HRs of 23.230, 8.037, 132.589, and 41.823 for FE exudative MNV occurrence, respectively. Hypopigmentation on CFP and SIRE on OCT could represent the same lesion. Even small hypopigmentation and small RPE elevation were significant risk factors for progression to exudative MNV.

## Introduction

Age-related macular degeneration (AMD) is the leading cause of blindness in developed countries^1^. Multiple epidemiologic studies and clinical trials have evaluated phenotypic risk factors amongst which large, central drusen and pigmentary abnormalities have been implicated as important risk factors for the disease progression to late AMD^2–6^.

The vascular endothelial growth factor (VEGF) inhibitors administered for the treatment of exudative macular neovascularization (MNV) secondary to AMD has curtailed the rate of legal blindness^7,8^. However, early treatment of exudative MNV is critical to improve the prognosis and prevent the permanent loss of central vision^9^. Hence, close surveillance is of paramount importance in susceptible individuals with high-risk factors for exudative MNV.

The Age-related Eye Disease Study (AREDS) simplified severity scale (AREDS Report No. 18)^6^, assigns the same point for the presence of one or more large (≥ 125 µm) drusen and the presence of any pigmentary abnormalities on color fundus photography (CFP) in each eye. According to the AREDS severity scale (AREDS Report No. 17)^5^, depigmentation larger than 0.5 disc area (DA) showed the highest 5-year incidence of neovascular AMD among all scales of drusen area and pigmentary abnormalities. Pigmentary abrnormalities have as much clinical significance as drusen and deserve more attention.

The prevalence of drusen in Asian patients with exudative AMD may be lower than in Caucasian patients with exudative AMD^10,11^. In a recent study regarding the fellow eyes of unilateral exudative AMD patients, there were no drusen in the fellow eyes in 96 out of 280 patients (34.3%)^12^. In Asian patients with exudative AMD, up to half have been reported as polypoidal choroidal vasculopathy (PCV) and the prevalence of drusen has been known to be lower in PCV patients than in patients with typical AMD^13^. Although drusen is a hallmark of the disease and its’ most important feature, pigmentary abnormalities may be relatively more important in Asian patients with AMD. To further clarify the clinical significance of pigmentary abnormalities itself, we investigated the progression to late AMD in the fellow-eyes without significant drusen of those patients with unilateral exudative MNV and long-term follow-up.

## Methods

### Patients

A retrospective study was conducted on patients with newly diagnosed and treatment-naïve exudative MNV in the Department of Ophthalmology at Yonsei Medical Center between January 2010 and June 2016. All consecutive patients were selected for medical record review. This study was approved by the Institutional Review Board (IRB) at Yonsei University Medical Center (IRB number: 4-2020-0276). Informed consent was waived due to the retrospective nature of the study, and the waiver was provided by the IRB. All study protocols adhered to the tenets of the Declaration of Helsinki.

We have analyzed patients with unilateral exudative MNV in the first eye and no signs of “significant drusen” and late AMD (defined as exudative MNV or any geographic atrophy) in the FE, and those who underwent more than 3 years of follow-up.

“Significant drusen” was defined by the presence of at least one large (≥ 125 µm) or numerous medium drusen (drusen number ≥ 20, 125 µm > drusen size ≥ 63 µm) at the central macula [within 1 disc diameter (DD) of the fovea] and/or subretinal drusenoid deposits (SDD). The presence of drusen and SDD was determined using CFP, infra-red reflectance (IR) image and OCT. SDDs were diagnosed when ≥ 10 discrete whitish deposits were observed on CFP and/or IR images; these deposits corresponded to material accumulated in the subretinal space on OCT images.

All patients underwent anti-VEGF therapy (Ranibizumab, Aflibercept, Bevacizumab) for exudative MNV of the first eye. Patients had presented for follow-up at 1- to 3-month intervals, depending on the disease activity.

All the included patients had undergone a comprehensive ophthalmological examination at the initial presentation, which included measurement of the best-corrected Snellen visual acuity, slit-lamp biomicroscopy, indirect fundoscopy, color fundus photography (CFP; VISUCAM, Zeiss, Germany), fluorescein angiography (FA), indocyanine green angiography (ICGA) using the Heidelberg retinal angiography device (HRA-II; Heidelberg Engineering, Dossenheim, Germany), and spectral-domain optical coherence tomography (SD-OCT; Spectralis; Heidelberg Engineering, Heidelberg, Germany)—including enhanced depth imaging (EDI). The protocol of SD-OCT consisted of 6-mm horizontal raster scans with 30–60-μm spacing that covered a 1500-μm diameter centered on the fovea. At every follow-up, patients underwent all examinations except for the angiographies and in cases of suspected exudative MNV of the FE showing newly developed subretinal fluid or intraretinal fluid on OCT, the occurrence of the exudative MNV was confirmed by additional FA and ICGA.

The subtype of exudative MNV was comprehensively diagnosed on the basis of findings from fundoscopy, angiography, and OCT. The diagnosis of PCV was based chiefly on ICGA findings, including polypoidal structures at the borders of the branching choroidal vascular networks^14^.

The patients were excluded if any of the eyes exhibited the following signs: choroidal neovascularization (CNV) secondary to other macular disorders such as angioid streaks; a refractive error of > 6.0 diopters; amblyopia; significant media opacities; a history of vitreo-retinal disorders or surgeries; and signs of “significant drusen” and late AMD in the FE at presentation.

### Imaging analysis of color fundus photography

The definition of pigmentary abnormalities followed that of the AREDS report number 6^15^. The pigmentary abnormalities on CFP were determined at the central macula (within 1DD of the fovea). Presence of geographic atrophy led to the exclusion of the case. Geographic atrophy was defined as a sharply demarcated, usually circular zone of partial or complete depigmentation of the retinal pigment epithelium, typically with exposure of underlying large choroidal blood vessels. Hypopigmentation (depigmentation) denoted areas of depigmented (atrophied) retinal pigment epithelium that did not meet the requirements for geographic atrophy: being less well defined (that is, having less sharp edges), less regularity in shape (such as less circular- or oval-shaped), and/or less severe (i.e., underlying choroid was less visible) than geographic atrophy. Hyperpigmentation (increased pigment) signified the presence of clumps of gray or black pigment in or beneath the retina.

Measurements of hypopigmentation area and disc area (DA) were carried out using Image J. The hypopigmentation area was converted to DA by dividing by the measurement of DA. When multiple lesions with various sizes were present simultaneously in one eye, the eye was classified as a group of the largest lesion.

### Imaging analysis of optical coherence tomography

The terminology “shallow irregular RPE elevation” (SIRE) was recently reported^16^ and is a more elaborately defined term of the widely used concept, “double-layer sign”^17–19^. “SIRE” is defined as RPE elevations with a greatest transverse linear dimension of 1000 µm or more, an irregular RPE layer with a height of predominantly less than 100 µm, and a nonhomogeneous internal reflectivity with characteristic features of the double-layer sign. Small RPE elevation was defined as shallow RPE elevations having a longest diameter of less than 1000 µm.

The determination of position, length, and height of RPE elevations was performed using the Early Treatment Diabetic Retinopathy Study (ETDRS) Grid and software tools embedded in the OCT machine. “Attenuation or disruption of RPE and/or ellipsoid zone (EZ)” was determined as an OCT feature of decreased signal or discontinuation of the RPE and/or EZ band. Discrete, well-circumscribed hyperreflective lesions within the neurosensory retina, defined as intraretinal hyperreflective foci (IHRF), had reflectivity at least as bright as the RPE band. The minimum size of 3 pixels was set for IHRFs, to differentiate from noise and retinal capillaries.

Altogether, abnormal findings on OCT were classified as the absence of RPE elevation, small RPE elevation, and SIRE according to the presence and size of RPE elevation; additionally, the presence of “attenuation or disruption of RPE and/or EZ” or IHRF was assessed.

Three independent examiners (J.L., H.R.K., S.H.B.) reviewed all images and determined the results of all variables. There was good agreement between the three examiners (over 90% for categorical values). Discrepancies between examiners for categorical values and differences over 30% between the quantitatively measured values were resolved by open adjudication, while provided with access to original images and data. If no consensus was reached, a final decision was made by the senior examiner (S.H.B) and the two closest values were averaged. Otherwise, all three measures were averaged.

### Statistical analysis

Time-to-event endpoint (exudative MNV occurrence of FE) was analyzed using the Kaplan–Meier method, and subgroups according to pigmentary abnormalities on CFP and abnormal findings on OCT were compared using the log-rank test. Factors likely to have an association in the univariate analysis (*P* < 0.20) were tested using multivariate analysis to identify independent factors associated with the occurrence using the Cox proportional hazard model. Assessment of collinearity between variables was determined using the variance inflation factor (VIF). Two models were constructed including each of the CFP findings (model 1) and OCT findings (model 2). Harrell's C-index was used to quantify and compare the predictive accuracy of both models^20^. Paired comparisons of the C‐indexes were performed using a bootstrap resampling procedure. The statistics were analyzed using SPSS software (IBM SPSS Statistics, Armonk, NY, USA) or R software (version 3.6.2). A *P* value of < 0.05 was considered statistically significant.

## Results

A total of 678 consecutive patients had newly diagnosed and treatment-naïve exudative MNV between January 2010 and June 2016. Among these patients, 241 cases with unilateral MNV and FE having no significant drusen and geographic atrophy, who underwent at least 3 years follow-up period, met the elibility criteria and were included for further analysis.

The summary of data from enrolled patients is presented in Table 1. The mean (± SD) follow-up duration was 71.9 ± 21.3 months. The numbers (%) of followed-up patients at 3 and 5 years were 241 (241/241 = 100.0%) and 152 (152/241 = 63.1%).

**Table 1:** Summary of data collected from the 241 enrolled patients.

Data type	Data
**Demographics**	
Age (yrs), mean (SD)	67.1 (8.3)
Female gender, no. (%)	91 (37.8)
FU Duration (months), mean (SD)	71.9 (21.3)
**Baseline characteristics**	
**Color fundus photography finding, no. (%)**	
Large hypopigmentation (≥ 0.5DA)	30 (12.4)
Small hypopigmentation (< 0.5DA)	57 (23.7)
Hyperpigmentation	39 (16.2)
Extramacular drusen (Outside 1DD of the center of the macula)	123 (51.0)
**OCT finding, no. (%)**	
SIRE (≥ 1000 µm in length)	30 (12.4)
Small RPE elevation (< 1000 µm in length)	26 (10.8)
Attenuation or disruption of RPE and/or EZ	53 (22.0)
IHRF	45 (18.7)
**NV Type of first eye, no. (%)**	
PCV, Type 1 MNV, Type 2 MNV, Type 3 MNV	116, 88, 37,0 (48.1, 36.5, 15.4, 0.0)
**Subfoveal choroidal thickness (µm), mean (SD)**	
First eye	328.5 (106.1)
Fellow eye	295.6 (103.7)
**Outcome, Late AMD, no. (%)**	
Exudative MNV	21 (8.7)
Geographic atrophy involving fovea	0 (0.0)

SD = Standard deviation; FU = Follow-up; NV = Neovascularization; PCV = Polypoidal choroidal vasculopathy; MNV = Macular neovascularization; DA = Disc area; DD = Disc diameter; SIRE = Shallow irregular RPE elevation; EZ = Ellipsoid zone; IHRF = Intraretinal hyperreflective foci; AMD = Age-related macular degeneration.

### Concordance between CFP and OCT findings (Table 2)

**Table 2:** Matching and concordance between the presence and size of hypopigmentation on color fundus photography and corresponding abnormal findings on optical coherence tomography.

CFP finding OCT finding	Large hypopigmentation (≥ 0.5DA)	Small hypopigmentation (< 0.5DA)	No significant findings	Total
SIRE (≥ 1000 µm in length) (with ; without “attenuation or disruption of RPE and/or EZ”)	22 (13; 9)	6 (1; 5)	2 (2; 0)	30
Small RPE elevation (< 1000 µm in length) (with ; without “attenuation or disruption of RPE and/or EZ”)	5 (5; 0)	21 (8; 13)	0	26
No significant findings	0	10	151	161
“Attenuation or disruption of RPE and/or EZ” only	3	20	1	24
Total	30	57	154	241

CFP = Color fundus photography; OCT = Optical coherence tomography; SIRE = Shallow irregular RPE elevation; EZ = Ellipsoid zone;

kappa = 0.759, *P* < 0.001 (“Attenuation or disruption of RPE and/or EZ” only group was not included in the analysis, n = 217).

**Figure 1:**
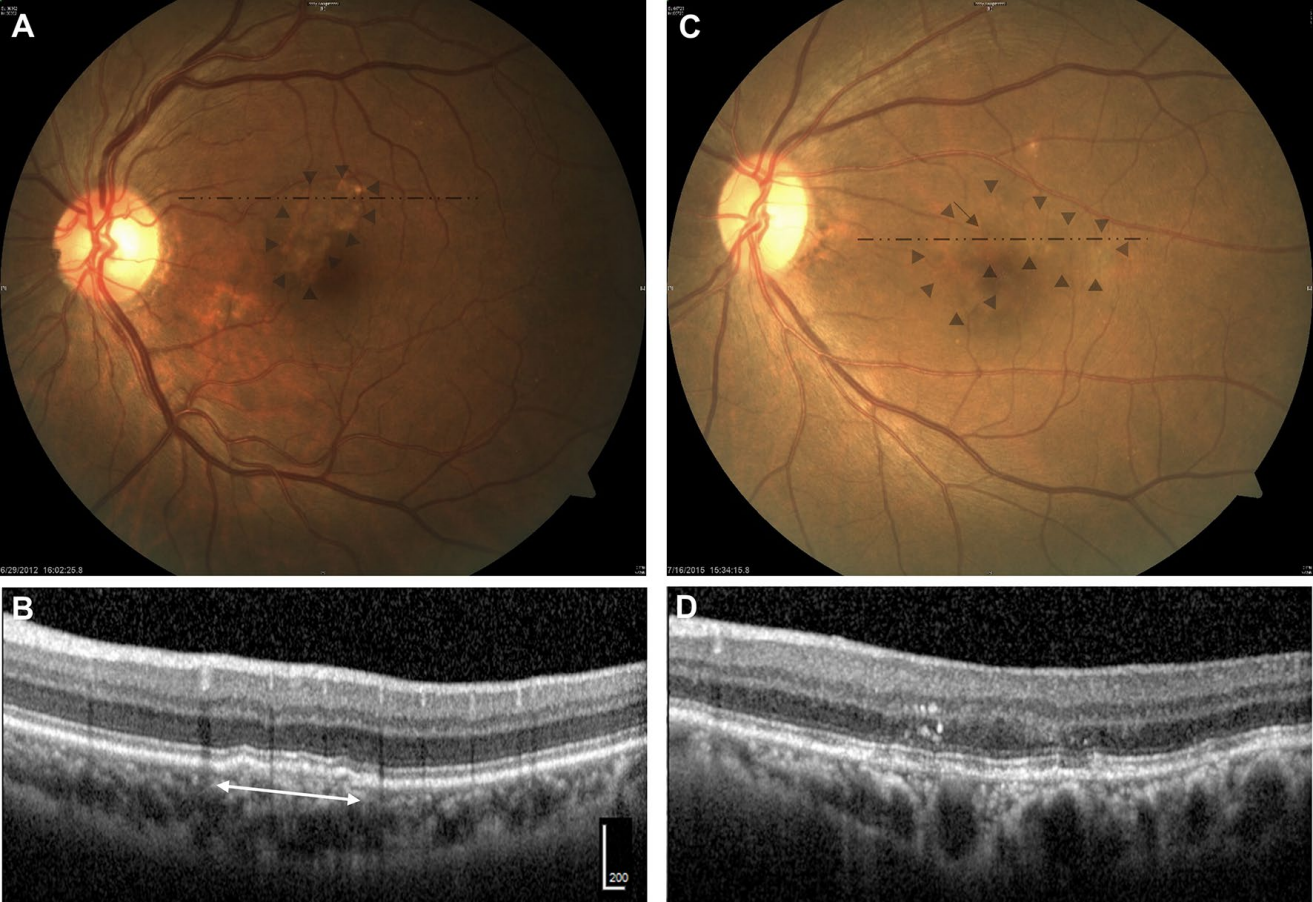
Representative types of pigmentary abnormalities on color fundus photography (CFP) and their corresponding optical coherence tomography (OCT) findings. (**A**) Large hypopigmentation (≥ 0.5 disc area; DA) on CFP. Well defined hypopigmented area is noted at the macula. The lesion size is 0.60 DA. The shape of the lesion is irregular and not circular or oval. Faint dash-dotted line indicates the position of OCT cross-section shown in (**B**). Faint triangles indicate the margin of the hypopigmentation. (**B**) Shallow irregular RPE elevation (SIRE) (≥ 1000 µm in length) on OCT. A 1350 µm in length, shallow, irregular RPE elevation is noted. The internal reflectivity is nonhomogenous. (Scale bar 200 µm). (**C**) Mottled area showing hypopigmentation with multiple hyperpigmentation spots on CFP. Poorly defined, irregular, mildly hypopigmented atrophic area is noted. Multiple hyperpigmented spots are located within the hypopigmented area. Faint dash-dotted line indicates the position of OCT cross-section shown in (**D**). Faint triangles indicate the margin of the hypopigmentation and faint arrow indicates the hyperpigmentation spot. (**D**) “Attenuation or disruption of RPE and/or ellipsoid zone” with intraretinal hyperreflective foci (IHRF). Multiple discontinuation and attenuation of the signal of ellipsoid zone band are observed. The attenuation of RPE band signal is noted. IHRF by RPE migration-up which corresponds to the location of the hyperpigmented spot on CFP is observed on OCT.

When confirming co-localization, hypopigmentation on CFP corresponded to SIRE and/or “attenuation or disruption of RPE and/or EZ” on OCT. All hyperpigmentation was identified as IHRF on OCT. Almost all SIRE on OCT manifested as hypopigmentation on CFP (28/30;93.3%). Representative images are depicted in Fig. 1. Regarding the presence and size of hypopigmentation on CFP and RPE elevation on OCT, there appears to be high agreement when comparing the concordance (kappa = 0.759, *P* < 0.001).

The diagnostic performance of CFP for detecting abnormal findings on OCT was favorable. Its sensitivity, specificity, positive predictive value (PPV), and negative predictive value (NPV) were 96.3% (77/80), 93.8% (151/161), 88.5% (77/87), and 98.1% (151/154), respectively.

### Incidence of fellow eye late age-related macular degeneration (Fig. 2)

No FE had progressed to geographic atrophy involving the fovea (0.0%) and the 3-year and 5-year incidences of FE exudative MNV were 5.4% and 8.6%, respectively.

**Figure 2:**
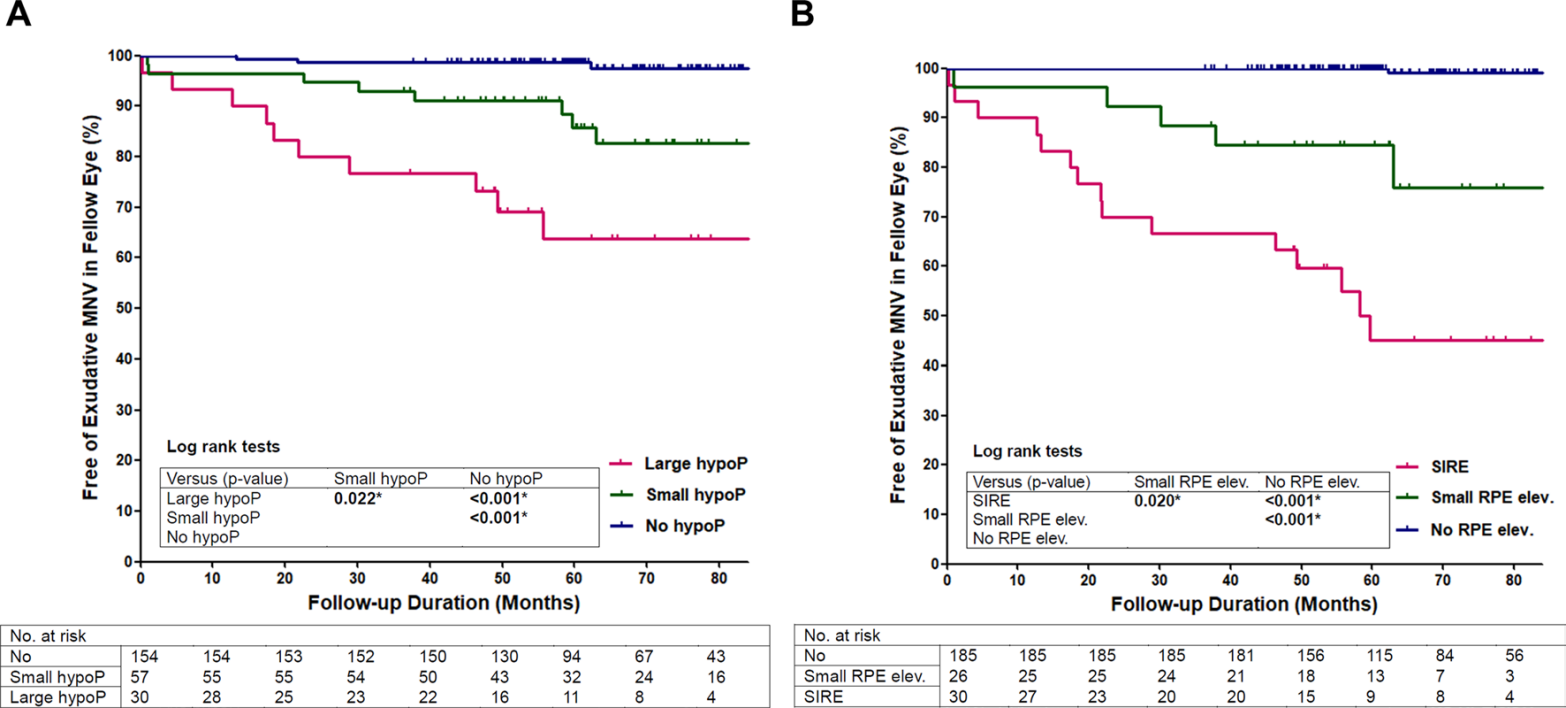
Time to development of exudative macular neovascularization (MNV) in the fellow eye without significant drusen and geographic atrophy in unilateral exudative MNV patients according to (**A**) Presence and size of hypopigmentation on color fundus photography (CFP), (**B**) Presence and size of RPE elevation on optical coherence tomography (OCT). The timepoint “0” is the timepoint at which MNV in the first eye has been diagnosed for the first time. The occurrence of exudative MNV in the fellow eye was used as an endpoint. The differences in survival between subgroups were displayed as *P* value by log-rank tests. HypoP, Hypopigmentation; SIRE, shallow irregular RPE elevation; elev., elevation.

The 5-year incidence of FE exudative MNV in the large hypopigmentation (≥ 0.5DA) and small hypopigmentation (< 0.5DA) groups on CFP were 36.2% and 14.2%, which were significantly higher than the no hypopigmentation group (vs. 1.3%; *P* < 0.001, < 0.001, Log-rank test; Fig. 2A). The 5-year incidence of FE exudative MNV in the SIRE (≥ 1000 µm in length) and small RPE elevation (< 1000 µm) groups on OCT were 55.0% and 15.6%, which were significantly higher than the no RPE elevation group (vs. 0.0%; *P* < 0.001, < 0.001, Log-rank test; Fig. 2B).

The 5-year incidence of FE exudative MNV in the hyperpigmentation group on CFP, “attenuation or disruption of RPE and/or EZ”, and IHRF groups on OCT were 21.3%, 23.2%, and 22.3%, respectively, which were significantly higher than the groups without these findings (vs. 6.1%, *P* = 0.002; vs. 4.5%, *P* < 0.001; vs. 5.5%, *P* = 0.002, Log-rank test; Supplementary Fig. 1). There were no differences in the incidence of FE exudative MNV according to the MNV subtype of the first eye and the presence of extramacular drusen (Supplementary Fig. 2).

### Clinical findings predicting the occurrence of exudative MNV in the FE

Results of the univariate analysis demonstrated that hypopigmentation on CFP, hyperpigmentation on CFP, RPE elevation on OCT, “attenuation or disruption of RPE and/or EZ” on OCT, and IHRF on OCT showed a significantly higher risk for the occurrence of exudative MNV in the FE (*P* < 0.001, = 0.004, < 0.001, = 0.001, = 0.003, respectively).

Two multivariate Cox proportional hazard models were constructed including each of the CFP and OCT findings. The two models revealed that hypopigmentation on CFP and RPE elevation on OCT were the only and most significant factors (*P* < 0.001 and < 0.001). Compared with no hypopigmentation, large hypopigmentation and small hypopigmentation on CFP showed HRs of 23.230 (95% CI 5.426–99.454, *P* < 0.001) and 8.037 (95% CI 2.088–30.928, *P* = 0.002). Compared with no RPE elevation, SIRE and small RPE elevation on OCT showed HRs of 132.589 (95% CI 16.562–1061.463, *P* < 0.001) and 41.823 (95% CI 4.711–371.261, *P* < 0.001).

In multivariate models, hyperpigmentation on CFP, “attenuation or disruption of RPE and/or EZ”, and IHRF on OCT were not shown to be significant risk factors (HR = 0.654, 95% CI 0.232–1.843, *P* = 0.422; HR = 1.186, 95% CI 0.453–3.102, *P* = 0.729; HR = 0.624, 95% CI 0.239–1.627, *P* = 0.335, respectively).

The predictive capacity, using Harrell's C-index (95% CI), was 0.851 (0.777–0.918) for the model with CFP findings (Table 3), 0.937 (0.902–0.966) for the model with OCT findings (Table 4) and the difference between both models was statistically significant (*P* = 0.0124).

**Table 3:** Cox proportional hazard model using color fundus photography findings for occurrence of fellow eye exudative macular neovascularization in patients with unilateral exudative macular neovascularization and fellow eye without significant drusen.

Characteristics	Occurrence of exudative MNV in fellow eye				
	Univariate analysis		Multivariate analysis		
	HR (95% CI)	*P*	HR (95% CI)	*P*	VIF
Age, year	1.038 (0.986–1.094)	0.156	1.028 (0.964–1.096)	0.406	1.103
**Sex**					
Male	0.993 (0.412–2.396)	0.987			
Female	1				
**MNV subtype**		0.533			
Type 1 MNV	1.686 (0.665–4.272)	0.271			
Type 2 MNV	1.165 (0.309–4.390)	0.822			
PCV	1				
**Drusen type**					
Extramacular drusen	1.585 (0.657–3.825)	0.305			
No drusen	1				
**CFP finding**					
Hypopigmentation		< 0.001*		< 0.001*	1.817
Large hypoP	21.683 (5.954–78.969)	< 0.001*	23.230 (5.426–99.454)	< 0.001*	
Small hypoP	7.664 (2.033–28.892)	0.003*	8.037 (2.088–30.928)	0.002*	
No hypoP	1		1		
Hyperpigmentation	3.684 (1.522–8.914)	0.004*	0.654 (0.232–1.843)	0.422	1.756
SubF CT of first eye, µm	1.002 (0.998–1.006)	0.266			
SubF CT of fellow eye, µm	1.003 (1.000–1.007)	0.084	1.002 (0.998–1.006)	0.275	1.096

Significant values are in bold.

MNV = Macular neovascularization; PCV = Polypoidal choroidal vasculopathy; CFP = Color fundus photography; hypoP = Hypopigmentation; SubF = Subfoveal; CT = Choroidal thickness; HR = Hazard ratio; CI = Confidence interval; VIF = Variance inflation factor.

*Significantly different.

**Table 4:** Cox proportional hazard model using optical coherence tomography findings for occurrence of fellow eye exudative macular neovascularization in patients with unilateral exudative macular neovascularization and fellow eye without significant drusen.

Characteristics	Occurrence of exudative MNV in fellow eye				
	Univariate analysis		Multivariate analysis		
	HR (95% CI)	*P*	HR (95% CI)	*P*	VIF
Age, year	1.038 (0.986–1.094)	0.156	1.016 (0.953–1.082)	0.635	1.121
**Sex**					
Male	0.993 (0.412–2.396)	0.987			
Female	1				
**MNV subtype**		0.533			
Type 1 MNV	1.686 (0.665–4.272)	0.271			
Type 2 MNV	1.165 (0.309–4.390)	0.822			
PCV	1				
**Drusen type**					
Extramacular drusen	1.585 (0.657–3.825)	0.305			
No drusen	1				
**OCT finding**					
RPE elevation		< 0.001*		< 0.001*	1.271
SIRE	128.283 (16.914–972.980)	< 0.001*	132.589 (16.562–1061.463)	< 0.001*	
Small	40.435 (4.721–346.289)	0.001*	41.823 (4.711–371.261)	0.001*	
No	1		1		
“Attenuation or disruption of RPE and/or EZ”	4.289 (1.820–10.103)	0.001*	1.186 (0.453–3.102)	0.729	1.793
IHRF	3.659 (1.539–8.701)	0.003*	0.624 (0.239–1.627)	0.335	1.851
SubF CT of first eye, µm	1.002 (0.998–1.006)	0.266			
SubF CT of fellow eye, µm	1.003 (1.000–1.007)	0.084	1.002 (0.998–1.006)	0.419	1.108

Significant values are in bold.

MNV = Macular neovascularization; PCV = Polypoidal choroidal vasculopathy; OCT = Optical coherence tomography; RPE = Retinal pigment epithelium; SIRE = Shallow irregular RPE elevation; EZ = Ellipsoidal zone; IHRF = Intraretinal hyperreflective foci; SubF = Subfoveal; CT = Choroidal thickness; HR = Hazard ratio; CI = Confidence interval; VIF = Variance inflation factor.

*Significantly different.

## Discussion

One of the main results of this study was that the hypopigmentation seen on CFP corresponded to SIRE and/or “attenuation or disruption of RPE and/or EZ” on OCT. The hypopigmentation on CFP and the RPE elevation on OCT significantly increased the risk of exudative MNV development, and this was in proportion to the size of the change.

It has been noticed that hypopigmentation showed a high risk for neovascular AMD by previous studies^5,21^. In Sarks’ report^21^ about the clinical and histological finding of the senile eye with clinically unsuspected CNV, large depigmentation in the macula showed the highest sensitivity and specificity for sub-RPE neovascularization among clinical appearances including drusen with or without pigment mottling and other pigment disturbances such as fine or coarse pigment clumping. On AREDS Report No. 17^5^, a 9-step severity scale that combines a 6-step drusen area scale with a 5-step pigmentary abnormality scale was developed. Among them, depigmentation larger than 0.5 DA showed the highest 5-year incidence of 22.9% for progression to neovascular AMD. Smaller depigmentation (< 0.5 to ≥ 0.0056DA ) had a 5-year incidence of 13.0% for neovascular conversion. Although there were differences in the main ethnicity of enrolled patients and the exclusion of drusen between the AREDS and this study, the 5-year incidence of FE exudative MNV in those with the large hypopigmentation (≥ 0.5DA) and small hypopigmentation (< 0.5DA) were 36.2% and 14.2%, respectively, which were not much different to the results from the AREDS. Our results suggest that a significant portion of the large depigmentation in the AREDS would be subclinical MNV, which may appear as SIRE on OCT. These findings are of significant importance in the real-world clinical setting, as that would mean that SIRE on OCT would manifest as hypopigmentation on CFP or ophthalmoscopic examination.

Subclinical type 1 MNV have a higher annual risk of exudation compared to eyes without these lesions^19^. Previous studies have shown that the “double-layer sign” on structural OCT corresponds to non-exudative type 1 MNV on OCTA^19,22,23^. A recent article by Shi et al. demonstrated that the double-layer sign on OCT showed good predictive values for detecting NE-MNV as confirmed by OCTA^24^. Narita et al. recently reported structural OCT signs suggestive of NE-MNV, which were called SIRE^16^. We adopted the standard of 1000 µm in length for the larger size as described by Narita et al.^16^. We confirmed that the 5-year incidence of FE exudative MNV in the SIRE group (≥ 1000 µm) was as high as 55.0%, despite the absence of significant drusen.

In this study, even small-sized hypopigmentation or RPE elevation showed a significant risk for exudative MNV with long-term follow-up. According to a previous report about the natural history of subclinical MNV^19^, small RPE elevation could be identified at the site of exudation where the MNV developed. The authors described that these small RPE elevations were the first sign of type 1 MNV and can serve as harbingers of impending exudation, and our results support their findings. Small RPE elevation is difficult to distinguish from drusen on OCT. Since we excluded patients with drusen on CFP, small RPE elevation on OCT seems to appear as hypopigmentation rather than typical drusen in CFP. This may be characteristic of vascularized drusen^25^, and OCTA would help in distinguishing between them. Of course, there may be a mixture of small typical drusen that was not detected in the CFP. However, small RPE elevation on the OCT, which may appear as hypopigmentation and not drusen, seems to be a risk factor for exudative AMD.

Hyperpigmentation and IHRF did not show significant risk in the multivariate prediction model after correcting for confounding factors. This result was different from reports that hyperreflective foci in OCT were the most important predictor of neovascular conversion in drusen accompanied cases^26^. In AMD, pigment migration can typically occur when the RPE band is disrupted and RPE migrates up above large drusen and less commonly without drusen, such as in our study. Unlike IHRF accompanying large drusen, IHRF without drusen was considered to have no additional risk for neovascular conversion. Thus, IHRF also needs to be subdivided for its clinical significance.

**Figure 3:**
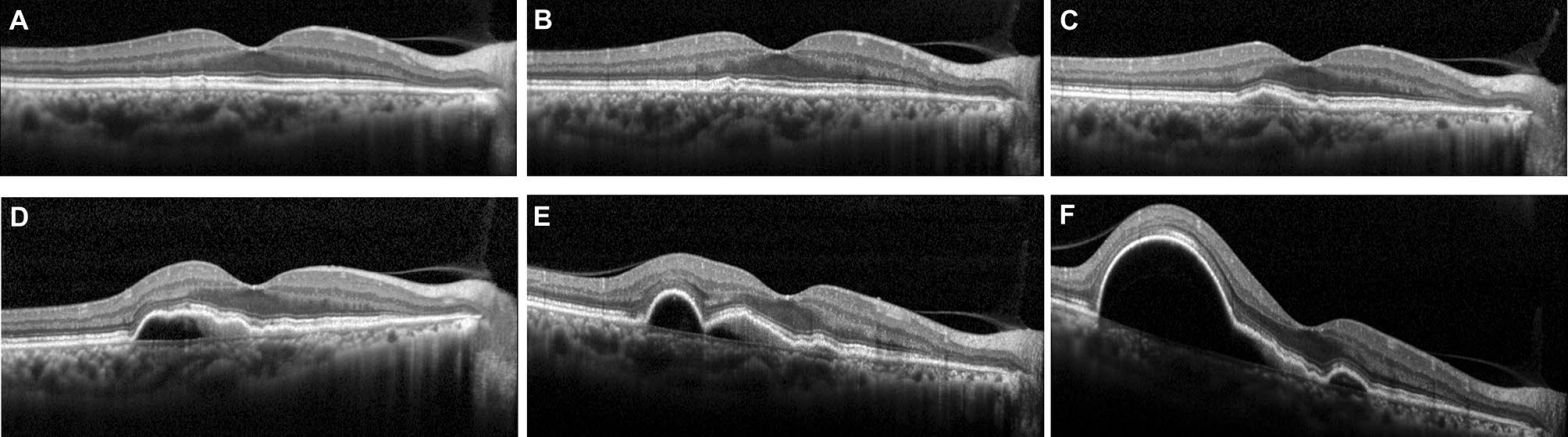
Even a small RPE irregularity or elevation may be an early precursor lesion of exudative macular neovascularization (MNV). This is a representative case of progression from small RPE irregularity or elevation to large polypoidal choroidal vasculopathy in 3 years and 6 months. Anti-vascular endothelial growth factor therapy had not been administrated until the occurrence of the subretinal fluid. All OCT series are horizontal section images of the same location across the center of the fovea. (**A**) Small RPE irregularity is noted with 620 µm length and temporal to the fovea. (**B**) One year after the first presentation, the small and shallow RPE elevation has been become more pronounced. The height and length of the lesion increased slightly. (**C**) At 1 year 9 months after the first presentation, double-layer sign with low to medium internal reflectivity is prominent and the height has considerably increased. (**D**) At 2 years and 2 months after the first presentation, the PED progressed and hyperreflective neovascularization underneath the RPE band is presumed. (**E**) At 3 years after the first presentation, the size of the lesion increased and polyp-like structure is seen. (**F**) At 3 years and 6 months after the first presentation, large polypoidal PED is formed.

In Asia, PCV is a major subtype of exudative MNV and accompanying drusen are less frequent^10,27^. Pachydrusen, which was noticed highly in association with PCV^10,28^, does not seem to increase the risk of late AMD and was considered to be a bystander of the pachychoroid phenotype rather than a precursor lesion^12^. As in type 1 MNV^19^, in PCV, small hypopigmentation or RPE elevation would be an early precursor lesion as depicted in the representative figure (Fig. 3). This observation made in the current study would be more important in clinics for Asian AMD patients who have a high prevalence of PCV and Type 1 MNV.

Even with a long follow-up period (mean 71.9 months), no central GA had occurred in our cohort. Depigmentation, which would be RPE elevation or atrophy, without drusen does not seem to be related with central GA. Our results are supported by a recent hypothesis in which RPE elevation corresponding to non-exudative type 1 MNV may act protectively against atrophy of the outer retina^29^. Another possible reason may be that almost all enrolled patients with “attenuated or disrupted RPE” were extrafoveal and did not invade the fovea. Thus, conventional central GA seems to be less related to pigmentary abnormalities and much more related to central soft drusen or SDD.

This study has the inherent limitations of a retrospective study and some risk factors, such as a history of smoking, genetic factors, and the use of an AREDS supplement, were not included. Because the characteristics and progression of AMD varies significantly according to ethnicity^27,30^, this study may not represent those of the general population. Future large-scale studies are required to reflect ethnicity and risk factors, including histories of smoking, AREDS supplementation, and genetic factors. In order to confirm that the CNV in the first eye is secondary to AMD, we tried to exclude myopic CNV and idiopathic CNV. Patients with refractive error greater than 6.0 diopters were excluded, and all enrolled patients were order than 55 years of age. However, this cannot guarantee that all enrolled patients definitely have CNV secondary to AMD and is a limitation.

Although the risk of large depigmentation for late AMD has been reported in the past, it has received relatively little attention. This study has shown the clinical significance of these lesions more clearly by showing that hypopigmentation corresponds to SIRE, an OCT finding of subclinical MNV recently gaining great attention. In addition, our results emphasize that the risk of progression to MNV increases with the size of hypopigmentation and RPE elevation, and even small hypopigmentation or RPE elevation warrant careful monitoring.

## Supplementary Information

Supplementary Information.

## Data Availability

All data generated or analysed during this study are included in this published article (and its Supplementary Information files). The datasets generated during and/or analysed during the current study are available from the corresponding author on reasonable request.
