# Supplementary material for: Pigmentary abnormality without significant drusen as a risk factor for late age-related macular degeneration
Source: Sci Rep. 2022 Jan 14;12:769. doi: 10.1038/s41598-022-04798-8 (PMC8760333; doi:10.1038/s41598-022-04798-8)

**Pigmentary Abnormality without Significant Drusen as a Risk Factor for Late Age-Related Macular Degeneration**

Junwon Lee, MD, PhD,^1^ Hyun Goo Kang, MD,^1^ Hae Rang Kim, MD,^2^ Christopher Seungkyu Lee, MD, PhD,^2^ Min Kim, MD, PhD,^1^ Sung Soo Kim, MD, PhD,^2^ Suk Ho Byeon, MD, PhD,^2,*^

^1^Department of Ophthalmology, Institute of Human Barrier Research, Gangnam Severance Hospital, Yonsei University College of Medicine, Seoul, South Korea

^2^Department of Ophthalmology, Eye Hospital, Severance Hospital, Institute of Vision Research, Yonsei University College of Medicine, Seoul, South Korea

***Corresponding author**:

Suk Ho Byeon, M.D., Ph.D.

Department of Ophthalmology, Institute of Vision Research, Yonsei University College of Medicine, Yonsei-ro 50-1, Seodaemun-Gu, Seoul, South Korea, 03722

Tel.: (+82) 2-2228-3570; Fax: (+82) 2-312-0541; E-mail: shbyeon@yuhs.ac; shbyeon@gmail.com

**Supplementary Contents**

Supplementary Figure 1.

Supplementary Figure 2.


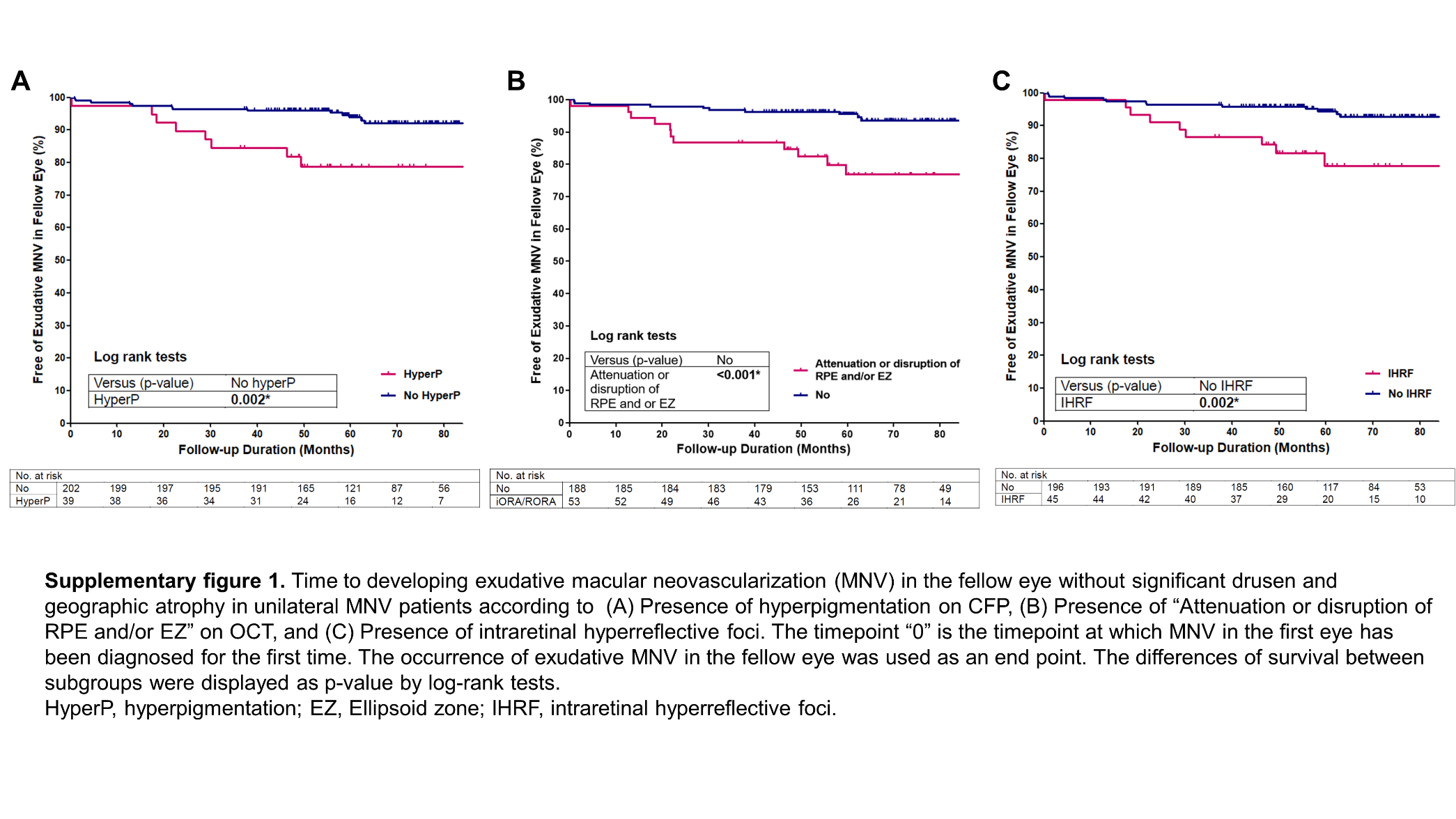


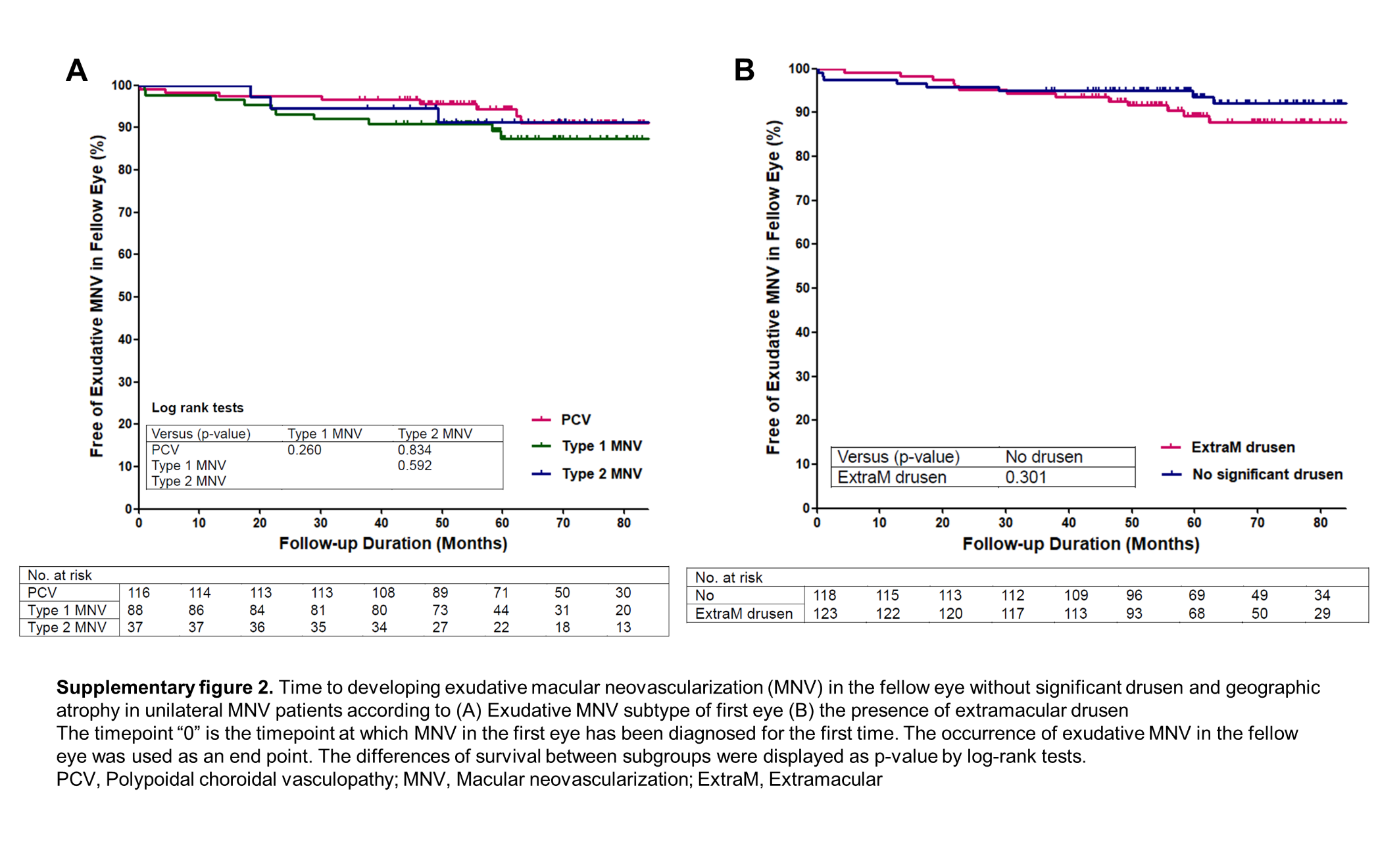

Supplement: Supplementary file 1 — Supplementary Information. [file 41598_2022_4798_MOESM1_ESM.docx]
